# Editorial: Large and Giant DNA Viruses

**DOI:** 10.3389/fmicb.2019.01608

**Published:** 2019-07-10

**Authors:** Jônatas Abrahão, Bernard La Scola

**Affiliations:** ^1^Laboratório de Vírus, Instituto de Ciências Biológicas, Departamento de Microbiologia, Universidade Federal de Minas Gerais, Belo Horizonte, Brazil; ^2^Microbes, Evolution, Phylogeny and Infection (MEΦI), Aix-Marseille Université UM63, Institut de Recherche pour le Développement IRD 198, Assistance Publique—Hôpitaux de Marseille (AP-HM), Marseille, France; ^3^Institut Hospitalo-Universitaire (IHU)—Méditerranée Infection, Marseille, France

**Keywords:** giant virus, large viruses, DNA virus, NCLDVs, evolution, pathogenesis

Since the seminal studies involving bacteriophages, the DNA viruses have fascinated the scientific community. DNA viruses were essential not only for the understanding of viral biological process, but also were a fundamental tool for the discovery and expanding knowledge related to cellular processes, such as transcription, translation, DNA repair, glycosylation and others. DNA viruses were also important characters during human history and evolution. The lethal and terrifying infection caused by a DNA virus, the smallpox disease caused by the variola virus, shaped and defined patterns of human migration, societies' interactions and raised innovative public health measures. In recent decades, some DNA viruses have been used as tools for heterologous protein expression and delivery, improving the field of vaccinology and diagnosis. In addition, some years ago, the discovery of the first mimiviruses shed new light on the study of DNA viruses field. Since then, many interdisciplinary studies, from distinct research groups, revealed breath-taking and controversial data regarding the origins, evolution and ecology of large and giant viruses. In this Research Topic, we received contributions from several colleagues on a broad range of topics related to large and giant DNA viruses.

Rodrigues et al. present a comprehensive meta-analysis of the currently known virosphere. In this study, it is crystal-clear that a substantial amount of knowledge on virology was obtained based on anthropocentric interests. The organisms with more viruses associated are human beings, plant crops, and domestic animals, revealing a huge gap on studies focused on viruses infecting species not related to humans. Contradicting this trend, we received many contributions on the discovery and biology of giant viruses that infect amoeba. A new and remarkable giant virus called Orpheovirus is described by Andreani et al. Orpheovirus is able to infect Vermoameba vermiformis and, with a genome exceeding 1.3 Mb and virions up to 1,300 nm in diameter, is one of the largest viruses described so far. Phylogenetic analysis provided evidence for a relationship between Orpheovirus and Pithovirus, however, some genetic characteristics revealed this new giant virus's divergent, independent evolution.

Silva et al. present an analysis of tupanvirus in *Vermoameba vermiformis*. Tupanvirus, a tailed giant virus, is the first to our knowledge that is able to infect more than one amoeba genus. In this paper, we learn that tupanvirus replication cycle in *V. vermiformis* is similar to tupanvirus cycle in *Acanthamoeba castellanii*. Outstanding scanning and electron microscopy images revealed fundamental steps of the cycle, including entry, factory formation, particle morphogenesis (including viral particle tail sprouting from factory), cell lysis and defective particles. The host-range of Marseillevirus (a virus discovered associated to *Acantamoeba*) was also explored by Aherfi et al. In this paper, the authors presented experimental inoculation of Marseillevirus in rats and mice models. Results revealed that, regardless the infection pathway utilized, Marseillevirus can be detected long-term in some organs, raising questions about the infective potential of this virus or a close relative in humans as suspected from cases of adenitis and lymphoma.

Evolutionary studies on giant virus were also explored in our Research Topic. Colson et al. performed a comprehensive study on the origins and ancestrality of giant viruses. By using phylogenetic and phenetic analyses, and the study of protein folding, to compare giant viruses and selected bacteria, archaea and eukaryota, the authors used their results to support the idea that giant viruses may cluster in a 4th branch of life, called 4th TRUC (for “Things Resisting Uncompleted Classifications”). This paper fuels the continuing (and perhaps controversial) debate on the origin of giant viruses. Chelkha et al. presented a phylogenomic study of the *Acanthameba polyphaga* draft genome, revealing more than 300 genes matching with viruses, including Pandoravirus, mimiviruses, Mollivirus sibericum, marseilleviruses, and Pithovirus sibericum. In a few case, genes seem to have been transferred from giant viruses to *A. polyphaga*, whereas in most of the cases the origins of those genes are equivocal. Assis et al. presented the genome characterization of the first two mimivirus of lineage C isolated in Brazil, called Mimivirus gilmour (MVGM) and Mimivirus golden (MVGD). In addition, the authors analyzed the pangenome of viruses belonging to *Mimivirus* genera, highlighting that discovery of new mimivirus isolates still contribute to the expansion of the pangenome and the consolidation of the core gene set. Aherfi et al. reported the isolation of three new Pandoravirus isolates, namely P. massiliensis, P. braziliensis, and P. pampulha. The authors presented an in-depth characterization of those isolates, including transcriptomics and genomics. In addition, the proteomics of P. massiliensis was described. The pangenome of the putative Pandoraviridae family was presented, revealing a large open pangenome and a small core genome. Louazani et al. analyzed the transcriptome of Faustovirus E12, presenting unexpected and complex splicing of the capsid gene. A total of 13 exons have been identified for the major capsid protein gene, including canonical and non-canonical splicing sites.

We also gathered new insights from papers focused on poxviruses. Cao et al. demonstrated the suppressive effect of resveratrol on vaccinia virus replication in various cell types. In this paper, authors suggest that resveratrol suppress the synthesis of viral DNA, affecting post-replicative gene expression. Szulc-Dabrowska et al. presented a comprehensive study on ectromelia virus, host immune response and viral evasion. Borges et al. presented serological evidence of silent (or possibly unreported) vaccinia virus exposure and disease in equids in southeast Brazil where the virus has been implicated in exanthematous outbreaks in cattle and humans.

The complex pathway of particle head assembly in the giant Salmonela phage SPN3US was explored by Ali et al. They presented data suggesting that a given prohead protease is able to cleave thousands of head proteins in just a few minutes to facilitate a major remodeling of the prohead prior to DNA packaging, impacting on viral assembly, final structure, composition and genome length. Huang et al. showed that the ubiquitin-proteasome system is important for replication of Singapore Grouper Iridovirus. Interestingly, several genes related to the ubiquitin-proteasome system were up/down-regulated during virus infection, and ubiquitin-proteasome system destruction impaired virus replication. Zhou et al. presented a new platform for genetic editing of Pseudoravies virus. The authors described the utilization of fosmid libraries for rapid generation of recombinant viruses. Xu et al. told us about the development of the recombinant immunotoxin called BoScFv-PE38, which has specific binding affinity for Bovine herpesvirus 1 glycoprotein D. They demonstrated that BoScFv-PE38 is internalized into MDBK cells compartments that inhibit BoHV-1 replication. Therefore, BoScFv-PE38 can potentially be employed as a therapeutic agent for the treatment of BoHV-1 infection. Finally, Zhang et al. presented data obtained *in vivo* suggesting that infection of chickens by infectious anemia virus can impair vaccinal immunity against Marek‘s disease.

## Author Contributions

All authors listed have made a substantial, direct and intellectual contribution to the work, and approved it for publication.

### Conflict of Interest Statement

The authors declare that the research was conducted in the absence of any commercial or financial relationships that could be construed as a potential conflict of interest.

